# The mechanoreceptor Piezo is required for spermatogenesis in *Bombyx mori*

**DOI:** 10.1186/s12915-024-01916-y

**Published:** 2024-05-20

**Authors:** Zhongjie Zhang, Xiaojing Liu, Bo Hu, Kai Chen, Ye Yu, Chenxin Sun, Dalin Zhu, Hua Bai, Subba Reddy Palli, Anjiang Tan

**Affiliations:** 1 Jiangsu Key Laboratory of Sericultural and Animal Biotechnology, School of Biotechnology, Jiangsu University of Science and Technology, Zhenjiang, 212100 China; 2 Key Laboratory of Silkworm and Mulberry Genetic Improvement, Ministry of Agriculture and Rural Affairs, Sericultural Scientific Research Center, Chinese Academy of Agricultural Sciences, Zhenjiang, 212100 China; 3 Department of Genetics, Development, and Cell Biology, Iowa State University, Ames, IA 50011 USA; 4 Department of Entomology, University of Kentucky, Lexington, KY 40546-0091 USA

**Keywords:** *Bombyx mori*, *Piezo*, Permatogenesis, Ale fertility

## Abstract

**Background:**

The animal sperm shows high diversity in morphology, components, and motility. In the lepidopteran model insect, the silkworm *Bombyx mori*, two types of sperm, including nucleate fertile eupyrene sperm and anucleate unfertile apyrene sperm, are generated. Apyrene sperm assists fertilization by facilitating the migration of eupyrene spermatozoa from the bursa copulatrix to the spermatheca. During spermatogenesis, eupyrene sperm bundles extrude the cytoplasm by peristaltic squeezing, while the nuclei of the apyrene sperm bundles are discarded with the same process, forming matured sperm.

**Results:**

In this study, we describe that a mechanoreceptor *BmPiezo*, the sole *Piezo* ortholog in *B*. *mori*, plays key roles in larval feeding behavior and, more importantly, is essential for eupyrene spermatogenesis and male fertility. CRISPR/Cas9-mediated loss of *BmPiezo* function decreases larval appetite and subsequent body size and weight. Immunofluorescence analyses reveal that BmPiezo is intensely localized in the inflatable point of eupyrene sperm bundle induced by peristaltic squeezing. BmPiezo is also enriched in the middle region of apyrene sperm bundle before peristaltic squeezing. Cytological analyses of dimorphic sperm reveal developmental arrest of eupyrene sperm bundles in *BmPiezo* mutants, while the apyrene spermatogenesis is not affected. RNA-seq analysis and q-RT-PCR analyses demonstrate that eupyrene spermatogenic arrest is associated with the dysregulation of the actin cytoskeleton. Moreover, we show that the deformed eupyrene sperm bundles fail to migrate from the testes, resulting in male infertility due to the absence of eupyrene sperm in the bursa copulatrix and spermatheca.

**Conclusions:**

In conclusion, our studies thus uncover a new role for *Piezo* in regulating spermatogenesis and male fertility in insects.

**Supplementary Information:**

The online version contains supplementary material available at 10.1186/s12915-024-01916-y.

## Background

The conversion of mechanical stimuli to electrochemical signals is essential for various physiological processes ranging from touch to proprioception and is associated with a number of diseases in humans [1, 2]. The evolutionarily conserved *Piezo* family has been identified as components of mechanically activated channels in numerous eukaryotes [3–5]. Vertebrates have two members of *Piezo*, *Piezo1* and *Piezo2*, whereas only one single homolog of *Piezo* was identified in plants and most invertebrates [6–8].

In mammals, *Piezo1* is mainly expressed in nonsensory cells or tissues [9, 10], whereas *Piezo2* is primarily expressed in sensory tissues that respond to touch [11–13]. In humans, dysfunction of *Piezo1/2* is closely related to multiple physiological disorders. Gain-of-function mutations in *Piezo1* are associated with dehydrated hereditary stomatocytosis [14–16]. Moreover, a common *Piezo1* gain-of-function mutation (E756el) causes red blood cell dehydration and attenuates *Plasmodium* infection in African populations [17]. A recent study further showed that individuals with *Piezo1* gain-of-function mutation (E756el) develop age-onset iron overload [18]. By contrast, loss-of-function mutations cause congenital lymphatic dysplasia [19, 20]. Gain-of-function mutations of *Piezo2* are related to distal arthrogryposis type 5, Gordon syndrome, and Marden–Walker syndrome [21, 22], while loss-of-function mutations result in scoliosis, impaired sensation of gentle touch, tactile pain, proprioception, and dysfunction of bladder control [23, 24].

In *Drosophila melanogaster*, null mutation of *DmPiezo* leads to severely reduced responses to noxious mechanical stimuli [8]. In addition, *DmPiezo* participates in the regulation of axon regeneration, feeding, mating behavior, and wound healing [25–30]. However, the physiological roles of *Piezo* in other insect species are still poorly understood.

In the current study, we investigate the physiological roles of *Piezo* in the lepidopteran model insect *Bombyx mori*. We knockout *BmPiezo* using the transgenic Cas9/sgRNA system. Disruption of *BmPiezo* caused reduced feeding amount and eupyrene spermatogenic arrest. These findings thus provide the first in vivo evidence that *Piezo* is essential for regulating insect growth and development, especially male reproductive development.

## Results

### Generation of BmPiezo mutant animals

Phylogenetic analysis of Piezo proteins revealed a high level of conservation among different species (Additional file 1: Fig. S1A). The three-dimensional structure of BmPiezo protein was predicted by the Phyre2 protein structure prediction online server [31], showing a similar structure with *Mus musculus* Piezo2 (Additional file 1: Fig. S1B-D). *BmPiezo* temporal and spatial mRNA expression was investigated by using q-RT-PCR. The result showed that *BmPiezo* was predominantly expressed in the epidermis, midgut, and genital glands (Additional file 1: Fig. S2A).

**Fig. 1:**
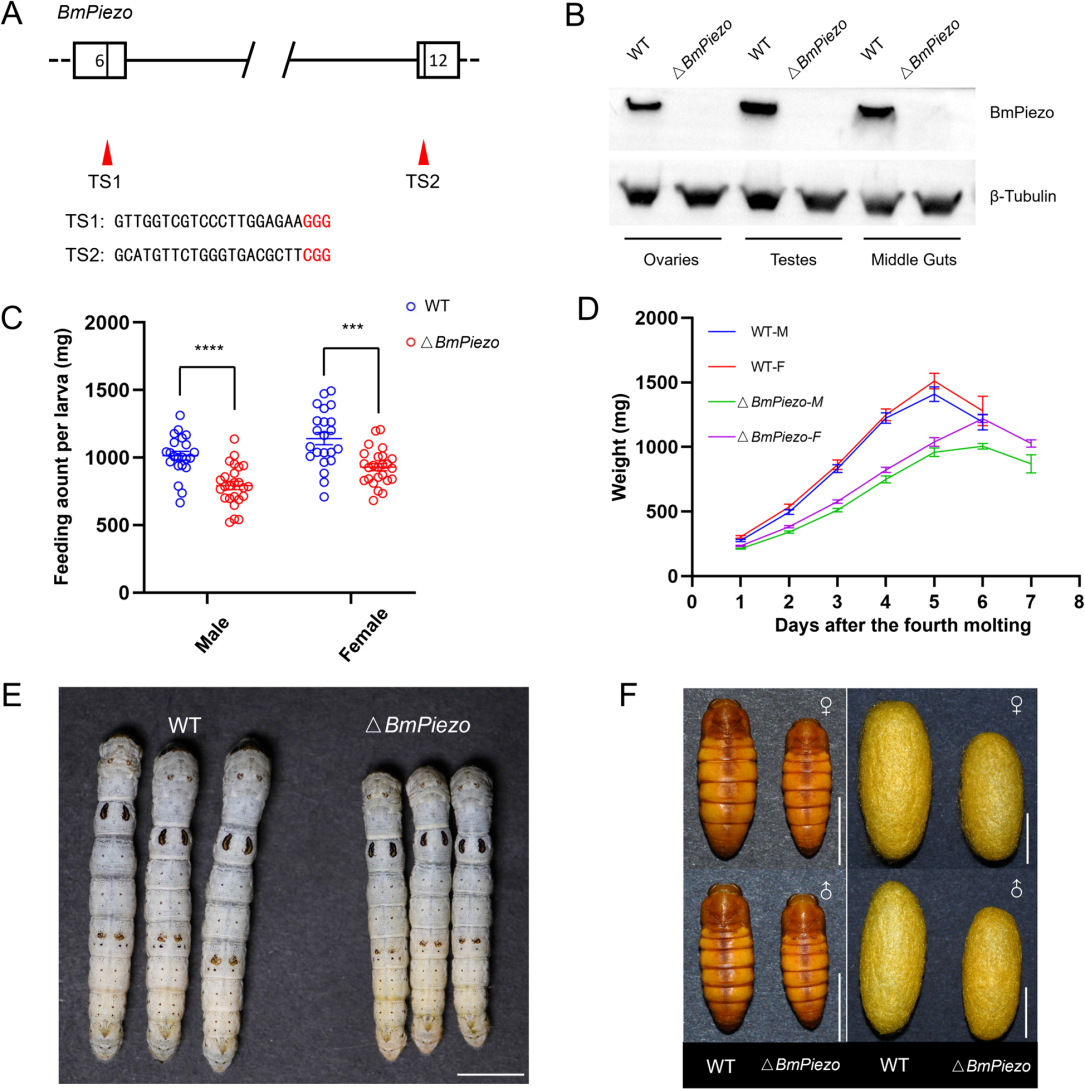
*BmPiezo* regulates *Bombyx* larval feeding. **A** Schematic diagram of the partial *BmPiezo* locus structure and two sgRNA-targeting sites, including TS1 and TS2. **B** Immunoblot analysis of BmPiezo protein in the testes from pupal stages day 7 and in the ovaries and midguts from day 4 of the fifth instar. The β-tubulin was used as a control. **C** The feeding dry weight of mulberry leaves by the fifth instar of WT and △*BmPiezo*. *n* 22 (WT-M), 22 (WT-F), 25 (△*BmPiezo*-M), and 25 (△*BmPiezo*-F), mean ± SEM, ****p < 0.0001 by two-tailed unpaired *t*-test, ***p < 0.0005 by two-tailed unpaired *t*-test with Welch’s correction. **D** Plot of the weight at a given time after the fourth ecdysis for the indicated genotype. *n* 20 (WT-M), 18 (WT-F), 25 (△*BmPiezo*-M), and 25 (△*BmPiezo*-F), mean ± SEM. **E** The larvae of day 4 of the fifth instar for the indicated genotype. Scale bar, 1 cm. **F** The upper graph shows male pupae and cocoons for the indicated genotype. The lower graph shows female pupae and cocoons for the indicated genotype. Scale bar, 1 cm

To explore the biological functions of *BmPiezo* in vivo, *BmPiezo* mutant lines (△*BmPiezo*) were established by using a binary transgenic CRISPR/Cas9 system described in our previous studies [32]. *BmPiezo* gene is located on chromosome 4 and is composed of 55 exons. Two sgRNAs targeting exon 6 and exon 12 were designed (Fig. 1A). △*BmPiezo* were generated by crossing the *BmPiezo-sgRNA* transgenic line with the *nos-Cas9* line. Genomic sequencing confirmed the somatic mutagenesis in the *BmPiezo* locus (Additional file 1: Fig. S2B). Western blotting analysis using an anti-BmPiezo antibody which directed against the C terminus of the protein revealed that no BmPiezo protein was detected in the ovaries, testes, and midguts of △*BmPiezo*, showing successful elimination of *BmPiezo* (Fig. 1B).

### BmPiezo regulates larval feeding behavior

Previous studies revealed that *DmPiezo* was essential for the regulation of feeding behavior in *D. melanogaster*. Activating *DmPiezo*-expressing enteric neurons decreases appetite, while *DmPiezo* knockout or *DmPiezo* neuron silencing increases food consumption [26–28]. To explore whether *BmPiezo* regulates feeding behavior in *B. mori*, newly molted fifth-instar wild type (WT) and △*BmPiezo* were fed with fresh mulberry leaves for 24 h, and the number of droppings was recorded. Compared to WT animals, the droppings of male and female △*BmPiezo* larvae decreased by 27.4% and 32.5%, respectively (Additional file 1: Fig. S2C). We also investigated the amount of intake food during the whole fifth larval instar and the result showed that the food intake of male and female △*BmPiezo* decreased by 21.7% and 18.6% respectively (Fig. 1C). The △*BmPiezo* larvae grew slower than WT during the fifth instar stage (Fig. 1D). Reduced food intake resulted in smaller larvae and pupae (Fig. 1E, F). The whole cocoon weight of male and female △*BmPiezo* decreased by 21.2% and 27.2%, respectively (Additional file 1: Fig. S2D). The pupal weight of male and female △*BmPiezo* also decreased by 20.6% and 26.7%, respectively (Additional file 1: Fig. S1E). The absence of *BmPiezo* caused reduced feeding amount and pupal weight without significant effect on development. The percentage of pupation did not change in △*BmPiezo* (Additional file 1: Fig. S2F). These data suggested that loss-of-*BmPiezo* function reduced larval food intake and subsequent biomass in the silkworm.

To further explore the reason for the abnormal feeding bahavior, we performed RNA-seq analysis by using midguts from the third day of the fifth instar in both WT and △*BmPiezo*. Differential expression analysis of RNA-seq analysis identified 238 differentially expressed genes, among which 92 genes were upregulated, and 146 were downregulated in △*BmPiezo*. Kyoto Encyclopedia of Genes and Genomes (KEGG) analysis revealed that ribosome biosynthesis upregulated significantly in △*BmPiezo* (Additional file 1: Fig. S3A). We found that the ribosomal protein genes including *BmRPL13*, *BmRPL26*, *BmRPL34*, *BmRPS13*, and *BmRPS24* were regulated in △*BmPiezo* (Additional file 1: Fig. S3B). We speculated that the upregulation of ribosome biosynthesis was a compensatory effect for reduced feeding amount to guarantee protein biosynthesis for silkworm growth and development.

### BmPiezo is required for male fertility

**Fig. 2:**
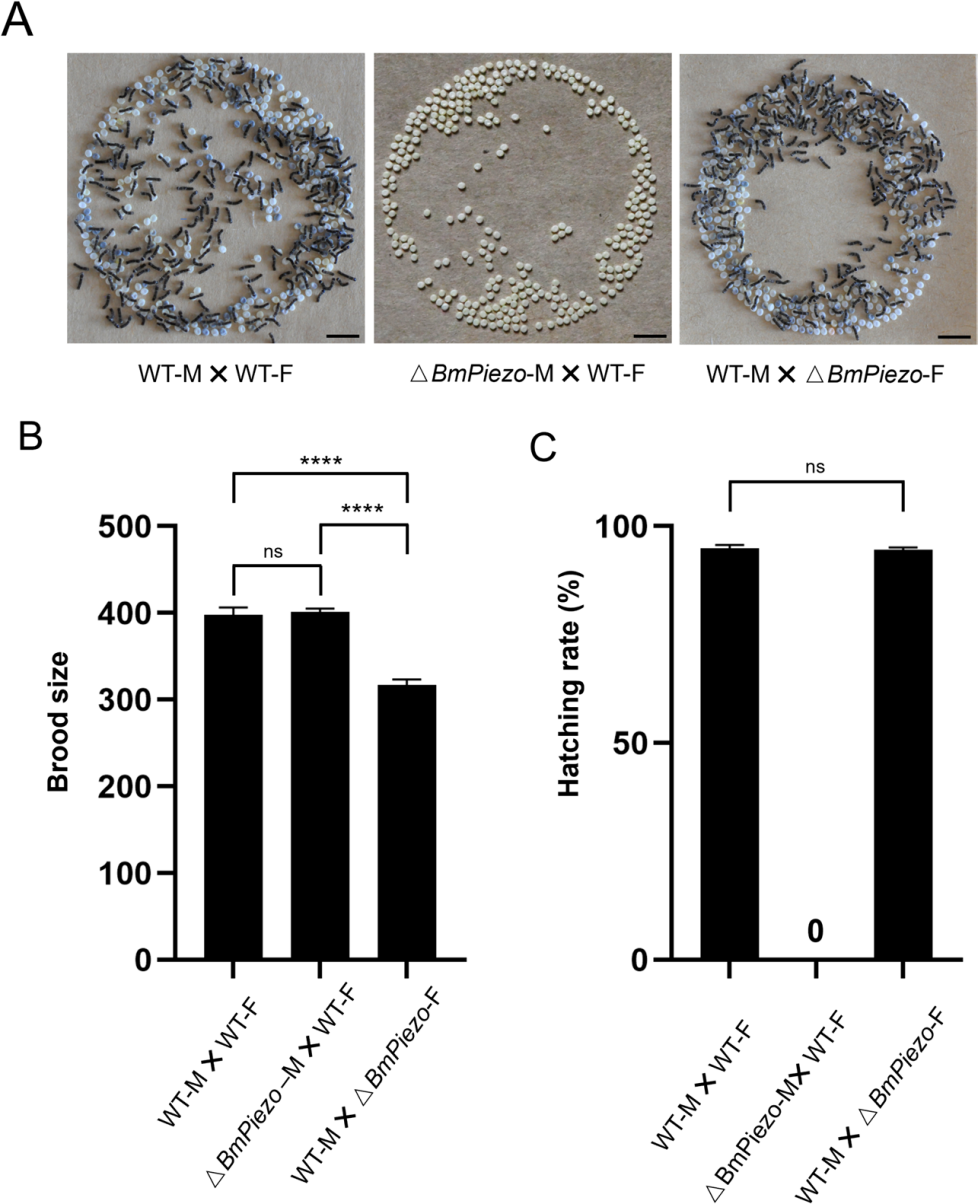
Loss of *BmPiezo* resulted in male infertility. **A** Representative photograph of eggs after 10 days laid by a wild-type female mated with wild type, wild-type female with △*BmPiezo* male and △*BmPiezo* female mated with wild type. Pale yellow eggs are unfertilized. **B** Brood sizes recorded from eggs laid in **A**. *n* 15 broods, mean ± SEM, ns: not significant, ****p < 0.0001 by Brown-Forsythe and Welch ANOVA with Dunnett’s T3 multiple comparisons test. **C** Analysis of the hatching rate. The results are expressed as percentages from 10 brood tests with two-tailed unpaired *t*-test. An index of 0% indicates the absence of newly hatched larvae

In *Caenorhabditis elegans*, the only *Piezo* ortholog of *Pizo-1* functions in different reproductive tissues of the hermaphrodite to ensure proper ovulation and fertilization [7]. Since *BmPiezo* is highly expressed in the genital glands (Additional file 1: Fig. S1A), we investigated that if loss-of-*BmPiezo* function affects *B. mori* fertility. Egg numbers laid by WT females mated with △*BmPiezo* males did not change significantly (Fig. 2A, B). However, the eggs laid by WT females mated with △*BmPiezo* males did not hatch (Fig. 2A, C). Compared to the egg number laid by WT female mated with WT male, egg numbers laid by △*BmPiezo* female mated with WT male decreased by 20.26% (Fig. 2B), and eggs hatched normally (Fig. 2A). We further confirmed that △*BmPiezo* males had no difference in the copulation behaviors including copulation success and copulation duration (Additional file 1: Fig. S4). These results suggested that *BmPiezo* is specifically required for male fertility.

### BmPiezo is indispensable for eupyrene spermatogenesis

**Fig. 3:**
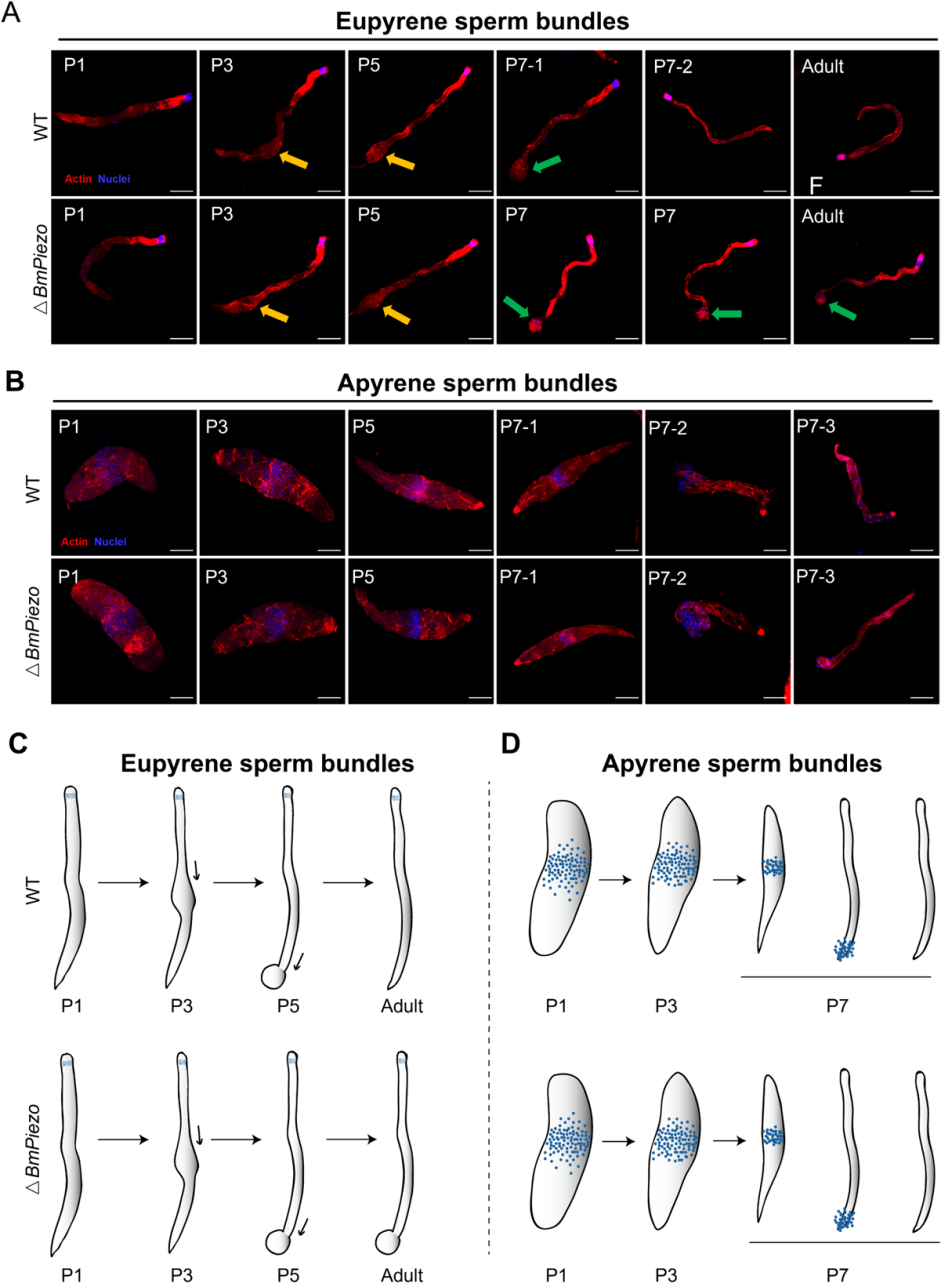
Loss-of-*BmPiezo* leads to developmental arrest of eupyrene sperm bundles. **A** Representative confocal images of eupyrene sperm bundles from WT and △*BmPiezo*. Orange arrows indicate the inflated position containing cytoplasmic debris caused by peristaltic squeezing. Green arrows indicate the posterior end of eupyrene sperm bundles with cytoplasmic debris after peristaltic squeezing. P1, pupal stages day 1. Blue, Hoechst; red, filamentous actin (F-actin). Scale bars, 50 µm. **B** Representative immunofluorescence images of apyrene sperm bundles of WT and △*BmPiezo* for the indicated stage. Blue, Hoechst; red, F-actin. Scale bars, 100 µm

To investigate the underlying mechanisms for the male sterility of △*BmPiezo*, we examined the status of spermatogenesis in △*BmPiezo* males. In WT animals, both eupyrene and apyrene spermatocytes undergo 2 successive meiotic divisions to produce 256 spermatids enveloped by the somatic cyst cells [33]. Following elongation and differentiation, the 256 spermatids develop into eupyrene or apyrene sperm bundles of 256 spermatozoa. The eupyrene and apyrene sperm bundles of silkworms have different morphology during spermiogenesis [33]. In WT animals, cytoplasmic debris was discarded from the posterior end of eupyrene sperm bundles from day 7 of the pupal stage (P7). In contrast, the cytoplasmic debris was enriched in the posterior end of eupyrene sperm bundles in △*BmPiezo* along with spermatogenesis and remained in the posterior end until the adult stage (Fig. 3A). In contrast, apyrene sperm bundles appeared to have normal morphology in △*BmPiezo*, with the distribution of small round micronuclei in the middle region day 1 of the pupal stage (P1) and sustaining round sperm nuclei in the middle of the bundles until being discarded by peristaltic squeezing during later pupal development (Fig. 3B).

**Fig. 4:**
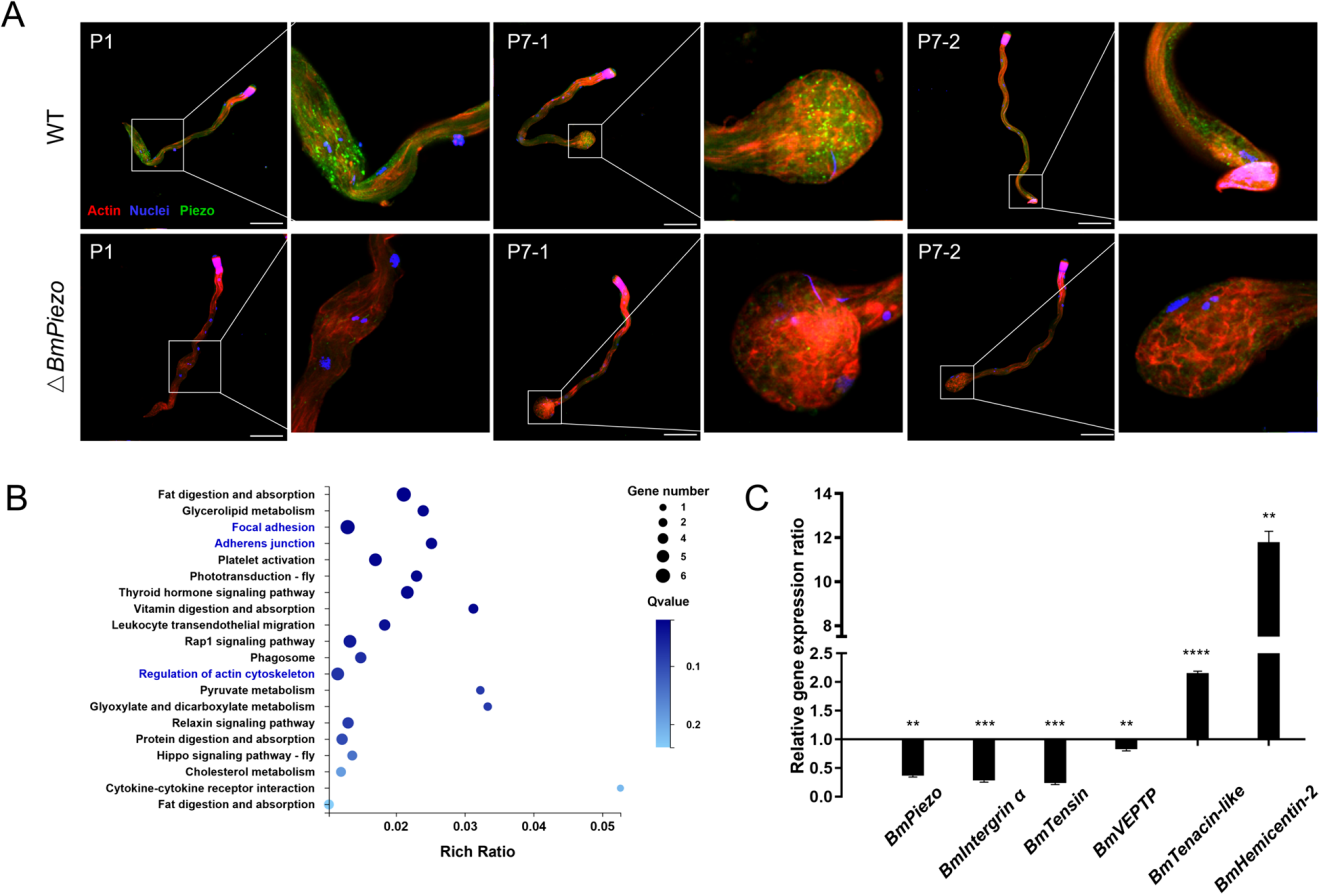
Loss-of-*BmPiezo* leads to dysregulation of the cytoskeleton in eupyrene sperm bundles. **A** Representative confocal images of eupyrene sperm bundles of WT and △*BmPiezo*. Blue, Hoechst; red, F-actin; green, anti-BmPiezo. Scale bars, 50 μm. **B** The top 20 enriched Kyoto Encyclopedia of Genes and Genomes (KEGG) pathways of DEGs with *p* < 0.05. Three significantly changed cytoskeleton-related pathways are in blue. **C** Validation of RNA-seq analysis revealed the gene expression changes in the three cytoskeleton-related pathways by q-RT-PCR. The results were measured in triplicate and are shown as mean ± SEM. The asterisks indicate the significant differences compared with the relevant control with a two-tailed unpaired *t*-test. *P < 0.05; **P < 0.01; ***P < 0.001, ****P < 0.0001

The localization of BmPiezo during spermatogenesis was investigated using immunofluorescence staining with anti-BmPiezo antibody. In WT animals, the expression level and location of BmPiezo changed dramatically in the eupyrene sperm bundle during the process of peristaltic squeezing. The BmPiezo was highly expressed in the inflated point of eupyrene sperm bundles induced by peristaltic squeezing from P3 to P7 (Fig. 4A). After peristaltic squeezing, the expression of BmPiezo declined sharply in mature eupyrene sperm bundle (Fig. 4A). The expression level and location of BmPiezo in apyrene sperm bundle also showed a dynamic pattern. Prior to the initiation of the peristaltic squeezing, BmPiezo is intensely localized in the middle region of apyrene sperm bundle (Additional file 1: Fig. S5). However, the expression of BmPiezo declined in squeezed and mature apyrene sperm bundles (Additional file 1: Fig. S5). In △*BmPiezo*, the expression level of BmPiezo decreased significantly in both eupyrene sperm bundles and apyrene sperm bundles (Fig. 4A, Additional file 1: Fig. S5). These results demonstrated that *BmPiezo* acted as a regulatory factor to ensure the elimination of cytoplasmic debris from the eupyrene sperm bundles.

### Dysregulation of the cytoskeleton in △BmPiezo

To further explore the molecular mechanisms of the △*BmPiezo* spermatogenic arrest, RNA-seq analyses were performed by using the testes from P7 pupae in both △*BmPiezo* and WT animals. Differential expression analysis of RNA sequences identified 63 differentially expressed genes, among which 22 genes were upregulated, and 41 were downregulated in △*BmPiezo* testes when compared to the WT groups. Kyoto Encyclopedia of Genes and Genomes (KEGG) enrichment analysis showed that focal adhesion, adherens junction, and actin cytoskeleton pathways were dysregulated in △*BmPiezo* (Fig. 4B). These 3 pathways are closely associated with actin cytoskeleton assembly and rearrangement. Actin filaments were reported to play vital roles in the peristaltic squeezing of eupyrene and apyrene sperm bundles in *B. mori* [34]. Focal adhesion is a large protein complexes that form between the extracellular matrix (ECM) and the cell [35]. Adherens junction is a form of cell–cell adhesion structure. *Integrin α*, *tensin*, and *vascular endothelial protein tyrosine phosphatase* (*VEPTP*) are key components of focal adhesion and adherens junction [36]. Integrins are cell surface receptors, which interact with the extracellular matrix and link to the actin cytoskeleton via an adaptor protein Tensin [35]. VEPTP have been shown to regulate cell–cell adhesion by regulating phosphorylation of the cadherin–catenin complex [36]. q-RT-PCR results showed that mRNA expression levels of *integrin α*, *tensin*, and *VEPTP* decreased by 27%, 24%, and 82%, respectively, in △*BmPiezo* compared to WT animals (Fig. 4C). In contrast, *tenascin-like* and *hemicentin-2* encoding proteins belong to the extracellular matrix protein family [37, 38], which were upregulated by 2- and 12-fold in △*BmPiezo* compared to WT animals (Fig. 4C). These results suggested that dysregulation of actin cytoskeleton assembly and rearrangement in △*BmPiezo* resulted in spermatogenesis arrest and male infertility.

### Migration of eupyrenesomen sperm bundles was disrupted in △BmPiezo

**Fig. 5:**
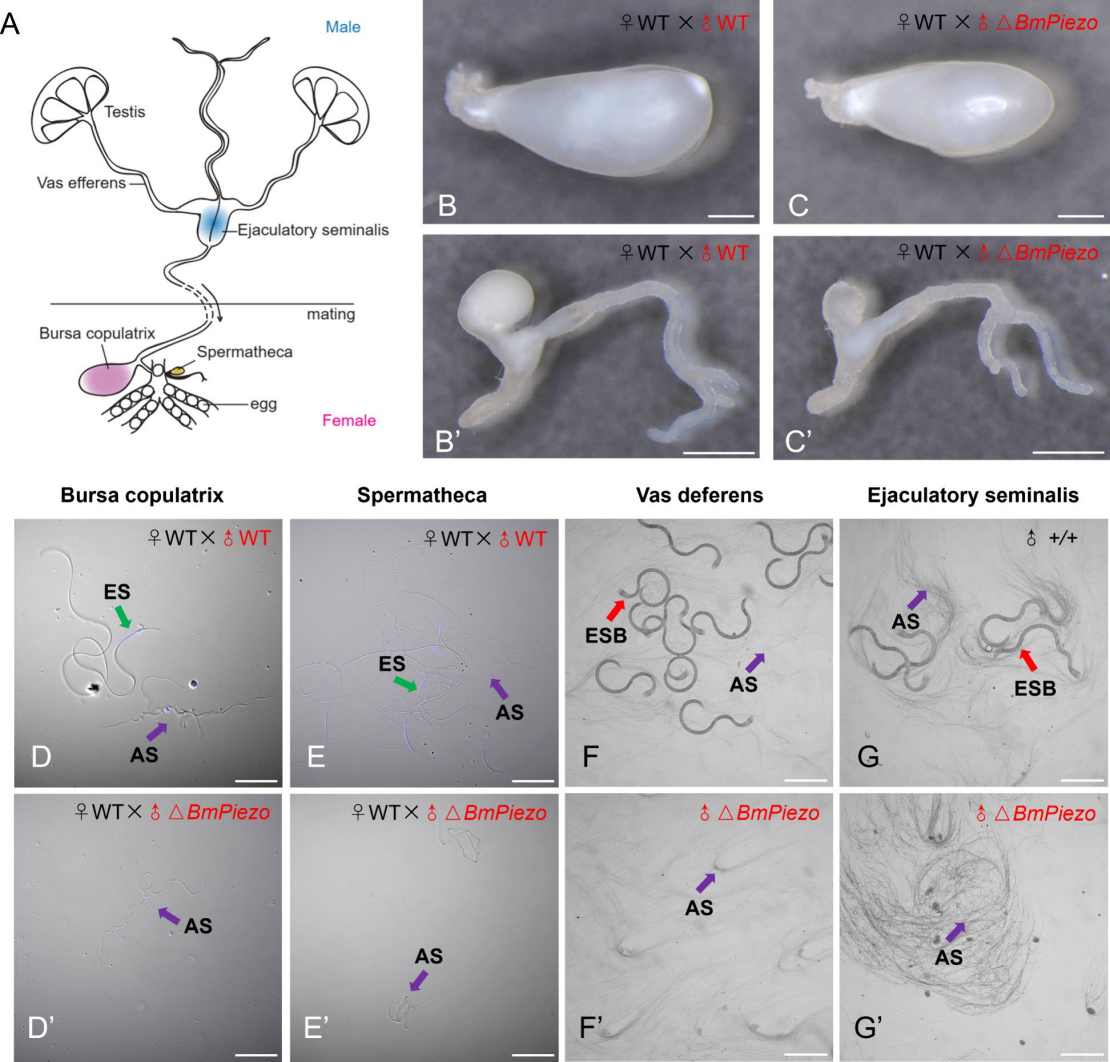
Loss-of-*BmPiezo* results in defective behavior of spermatozoa in adult male and female reproductive tracts. **A** Diagrams illustrating the male and female genital tracts modified from Chen et al.[40] **B**,** B’** Bursa copulatrix of females mated with WT and Δ*BmPiezo* males. **C**,** C’** Spermatheca of females mated with WT and Δ*BmPiezo* males. **D**,** D’** The smear of the bursa copulatrix. Blue, Hoechst. **E**,** E’** The smear of the Spermatheca. Blue, Hoechst. **F**,** F’** Eupyrene sperm bundles and apyrene spermatozoa in ejaculatory seminalis of unmated WT male and Δ*BmPiezo* male. **G**,** G’** Eupyrene sperm bundles and apyrene spermatozoa in the vas deferens of unmated WT male and Δ*BmPiezo* male. The purple and green arrows in **D**–**G** and **D’**–**G’** indicate apyrene and eupyrene sperm, respectively. The red arrow in **F** and **G** indicates eupyrene sperm bundle. Scale bars, 1 mm **B**, **B’**, **C**, **C’**, 50 µm **D**, **D’**, **E**, **E’**, and 200 µm **F**, **F**’, **G**, **G’**. ES, eupyrene sperm; AS, apyrene sperm; ESB, eupyrene sperm bundle

In *B. mori*, spermiation is initiated with the migration of apyrene sperm from the testes before eupyrene sperm bundles [39]. At the beginning of spermiation, apyrene sperm bundles shed their sheaths and liberate individual spermatozoa into the vas deferens. Subsequently, eupyrene sperm bundles migrated into the vas efferens and maintain the bundled state. Afterwards, apyrene spermatozoa and eupyrene sperm bundles migrate to the ejaculatory seminalis. During copulation, both types of sperm migrate from the male ejaculatory seminalis to the female spermatophore within the bursa copulatrix, where the eupyrene sperm bundles are fully dissociated while the apyrene spermatozoa acquire motility. Next, mobile apyrene spermatozoa facilitate eupyrene sperm migration to the spermatheca, where fertilization occurs (Fig. 5A).

To assess whether the migration of sperm is affected in △*BmPiezo*, we investigated the behavior of spermatozoa in the male and female reproductive tracts. The bursa copulatrix and spermatheca were full in WT females mated with WT males (Fig. 5B, C). The WT females mated with △*BmPiezo* males had full bursa copulatrix (Fig. 5B’), whereas their spermatheca was almost empty (Fig. 5C’). Both eupyrene and apyrene sperms were found in the bursa copulatrix and spermatheca of WT females and those mated with WT males (Fig. 5D, E, Additional file 2: Video S1, Additional file 3: Video S2). Only apyrene sperm was detected in the smears of the bursa copulatrix and spermatheca of WT females mated with the △*BmPiezo* males (Fig. 5D’, E’, Additional file 4: Video S3, Additional file 5: Video S4), indicating the absence of eupyrene sperm in their bursa copulatrix and spermatheca. Next, we examined the behavior of spermatozoa in the vas deferens and ejaculatory seminalis of unmated males. The vas deferens and ejaculatory seminalis of unmated WT males were full of apyrene spermatozoa and eupyrene sperm bundles (Fig. 5F, G). In contrast, the vas deferens and ejaculatory seminalis of unmated △*BmPiezo* contained only apyrene spermatozoa (Fig. 5F’, G’). These data suggested that the developmentally arrested eupyrene sperm bundles failed to migrate from the testes.

To further verify the functionality of apyrene sperm in △*BmPiezo*, double copulation experiments were performed using *BmSxl* mutants, which lack functional apyrene sperm but have functional eupyrene sperm [40]. We found that the double copulation rescued the infertility in △*BmPiezo* (Additional file 1: Fig. S6). This result confirmed that apyrene sperm sustained normal function in △*BmPiezo*.

## Discussion

The evolutionarily conserved Piezo family members are identified as components of mechanically activated channels [3]. The Piezo channels serve as mechanotransducers and govern fundamental physiological processes, such as stem cell differentiation, gastrointestinal transit, and sexual function [41–43]. In the present study, we identified a *Piezo* ortholog in *B. mori* and revealed that disruption of *BmPiezo* induced a decline of food intake during larval stages and defective eupyrene spermatogenesis which eventually resulted in male sterility.

In *C. elegans*, *Pizo-1* regulated not only the process of ovulation and fertilization but also food sensation, pharyngeal pumping, and defecation [7, 44]. Loss of long *Pezo-1* isoform expression resulted in increased pharyngeal pumping frequency and defecation frequency [44]. In mice, knockout of *Piezo2* in the dorsal root caused increased defecation frequency [42]. However, the *Piezo2*-mutant mouse showed normal food and water intake. Intriguingly, while it was reported that loss of *Piezo* in *Drosophila* increased both food consumption and body weight in the adult stage [27], *BmPiezo* knockout displays opposite phenotypes with reduced food intake and body weight (Fig. 1C, Additional file 1: Fig. S1D, E), showing functional diversity of *Piezo* in different insect species. The digestive system of insects shows high diversity in different species and in different developmental stages [45]. In *Drosophila*, *Piezo* knockout increased food consumption and body weight at the adult stage whereas no such analysis was performed at larval stages [26–28]. In *Bombyx*, *Piezo* knockout reduced the feeding amount at larval stages. These results indicated that *Piezo* may play different roles in insect food intake behavior. The prediction of the three-dimensional structure of BmPiezo, DmPiezo, and MmPiezo2 showed a similar structure. However, Piezo from *Bombyx*, *Drosophila*, and *Macaca* displayed a diverse role. These results suggested Piezo displayed diverse roles regardless of the similar structure in different species. Most lepidopteran insects including *B. mori* have dichotomous spermatogenesis which produces apyrene and eupyrene sperms. Numerous genes have been demonstrated to regulate *B. mori* spermatogenesis. Loss of *Maelstrom*, *protein arginine methyltransferase 5*, and *Vasa* functions disrupted both apyrene and eupyrene spermatogenesis [46, 47]. Knocking out *sex-lethal* disrupted apyrene sperm development [40, 48]. *Polyamine-modulated factor 1 binding protein* (*PMFBP1*), *poly(A)-specific ribonuclease-like domain-containing 1* (*PNLDC1*), and *Hua enhancer 1* (*Hen1*) were found to regulate the development of eupyrene sperm bundles [40, 49, 50]. These known defects of sperm bundles mainly affected the shape and the location of the cell nuclei. In contrast, we did not observe significant changes in the shape and location of the cell nuclei in △*BmPiezo*. Our results revealed that eupyrene sperm bundles of △BmPiezo were developmentally arrested at the late stage of spermatogenesis, suggesting that *BmPiezo* functioned in a novel regulatory mechanism governing dimorphic spermatogenesis in *B. mori*.

In the present study, we found that the cytoplasm of the eupyrene sperm bundles was enriched in the posterior end, presenting a spherical structure (Fig. 3A). The previous study showed that the eupyrene sperm bundles passed through the basement membrane between follicles and vas efferens during spermiation [39]. In △*BmPiezo*, immature eupyrene sperm bundles failed to migrate from the follicles to the vas efferens (Fig. 5F’), indicating that the inflated tail blocked the migration of immature eupyrene sperm bundles. Immunofluorescence staining revealed that BmPiezo was intensely localized in the posterior spherical structure of eupyrene sperm bundles induced by peristaltic squeezing (Fig. 4A), suggesting that BmPiezo is required for the last step of peristaltic squeezing. RNA-seq and qRT-PCR analyses demonstrated that spermatogenesis arrest in △*BmPiezo* was associated with focal adhesions, adherens junction, and regulation of actin cytoskeleton. Intriguingly, the actin cytoskeleton has been previously reported to contribute to the peristaltic squeezing of the sperm bundle in *B. mori* [34]. Application of cytochalasin D interrupted peristaltic squeezing [51]. Previous studies showed that various forms of mechanical stimulation, including external forces or endogenously originated local membrane tension can activate Piezo channels [2], and membrane tension is closely related to cell shape [52]. During the last step of peristaltic squeezing, the posterior end of the eupyrene sperm bundle forms a spherical structure. The dramatic change in the posterior end of eupyrene sperm bundle might be accompanied by changes in cell membrane tension. We hypothesize that the *BmPiezo* mutation resulted in a failure of membrane tension sensation, further disrupted the process of cell cytoskeleton assembly and rearrangement, and eventually affected the final release of cytoplasmic debris. Since eupyrene sperms and apyrene sperms show the diverse structure and biological functions, we presume that the process of peristaltic squeezing may be different in each kind of sperm, and different genes might be needed. Genes other than *Piezo* may participate in the transduction of mechanical stimulation during the peristaltic squeezing of apyrene sperms. The present study thus provides the first evidence that *Piezo* is essential for insect spermatogenesis. It will be interesting to explore whether *Piezo* has more functional roles in diverse physiological processes.

## Conclusions

Here, we describe that *Piezo*, a bona fide mechanoreceptor in mammals, plays key roles in nucleate fertile eupyrene spermatogenesis and male fertility in *B. mori*. Loss-of-function for *Piezo* induced food consumption decrease of silkworm larvae. More significantly, arrested eupyrene spermatogenesis in mutant silkworm males resulted in deformed eupyrene sperm bundles and failed to migrate from the testes. Our data provides the first in vivo evidence that a single mechanoreceptor regulates spermatogenesis in the animal kingdom including insects.

## Methods

### Silkworm strains

A multivoltine and non-diapausing silkworm strain, Nistari, was used in all experiments. Larvae were fed on fresh mulberry leaves under standard conditions at 25 °C and 75% relative humidity. Pupae were checked daily for emergence. Whole cocoon weight was counted at 4 days after pupation. One- to 2-day-old virgin males or females were used for fecundity tests.

### RNA isolation, cDNA synthesis, and quantitative real-time PCR (q-RT-PCR) analysis

Total RNA from the head, anterior silk gland, middle silk gland, posterior silk gland, epidermis, midgut, Malpighian tubule, fat body, testis, and ovary were isolated using TRIzol Reagent (Ambion) and treated with DNase I (Takara) to digest genomic DNA. cDNA was synthesized using the ReverAid First Strand cDNA Synthesis Kit (Thermo Fisher Scientific) following the manufacturer’s protocol. q-RT-PCR were performed using SYBR Green Real-time PCR Master Mix (Toyobo). The thermal cycling conditions were as follows: initial incubation at 95 °C for 5 min, 40 cycles of 95 °C for 15 s, and 60 °C for 1 min. Three independent biological replicates were performed in quantitative mRNA measurements, and data were normalized to *Bmrp49*. The primers used in q-RT-PCR are listed in Additional file 1: Table S1.

### Morphological investigation of sperm bundles and sperm

Sperm bundles or sperm from different stages were collected in 1.5-ml tubes and were fixed in PBS with 4% paraformaldehyde for 1 h. The samples were washed three times using PBS, then the actin proteins were stained with TRITC Phalloidin (1:200, YEASEN) for 1 h, and the nuclei were stained with Hoechst (1:200, YEASEN) for 10 min. The samples were washed three times using PBS, smeared on a microscope slide, and observed using the Nikon C2 Confocal Microscope.

### Immunofluorescence staining

BmPiezo antibodies (BmPiezo-R) were generated in rabbits using peptide-containing the amino acid residues (RPKEEPEEQRALPPSRSERS) at the end of the C-terminal of BmPiezo and affinity-purified at ABclonal. Immunofluorescence staining experiments were performed using sperm bundles isolated from excised testes. The collected sperm were fixed in permeabilizing buffer (1 × PBS + 4% paraformaldehyde + 0.1% Triton X-100) for 15 min, washed in PBST three times, and subsequently incubated in blocking solution (1 × PBS + 0.1% Triton X-100 + 1% bovine serum albumin) for 60 min. A primary antibody was added to the blocking solution, and then the samples were incubated at 4 °C for 36 h. After five washes in PBST, samples were incubated with the secondary antibody, TRITC Phalloidin, and Hoechst for 2 h at room temperature; washed five times with PBST; and subsequently mounted in PBS. All images were taken on a Nikon FV1000 microscope. Antibodies and dilutions used were as follows: BmPiezo-R (ABclonal), 1:100; FITC goat anti-rabbit IgG (H+L) (ABclonal, Cat. AS011), 1:100; and TRITC Phalloidin (YEASEN), 1:200.

### Silkworm germline transformation and CRISPR/Cas9-mediated construction of BmPiezo mutants

A binary transgenic CRISPR/Cas9 system was used to produce *BmPiezo* mutants. The transgenic silkworm, nos-Cas9 (*IE1-EGFP-nos-Cas9*) which expressed the Cas9 under the control of the *B.* mori *nanos* promoter (*nos*), was established [53]. To target the *BmPiezo* gene, the transgenic silkworm U6-sgRNAs (*IE1-DsRed2-U6-sgRNAs*) expressing *BmPiezo*-specific sgRNAs under the control of the U6 promoter was constructed as described previously [54]. The mixture of the transgenic plasmid and helper plasmids was injected into preblastoderm embryos (G_0_). G_0_ adults were sib-mated or crossed with wild type (WT) to obtain G_1_ progeny. Screening for transgenic lines carrying the DsRed2 marker was performed on newly hatched silkworms using a fluorescence microscope (Nikon AZ100, Japan). The nos-Cas9 lines and the U6-sgRNA lines were crossed to obtain *BmPiezo* mutants (△*BmPiezo*) with both EGFP and DsRed2 fluorescence markers. The primer sequences used for plasmid construction are listed in Additional file 1: Table S1.

### Genomic DNA extraction and mutagenesis analysis

Genomic DNA extracted from △*BmPiezo* at the adult stage was subjected to PCR amplification with *BmPiezo*-specific primers for mutagenesis analysis. Validation of *BmPiezo* knockout efficiency was conducted in the middle guts, testes, and ovaries of △*BmPiezo* by western blotting with the BmPiezo-R antibody (1:1000). Silkworm β-actin, detected by β-actin rabbit mAb (1:1000; ABclonal, Cat. AC015), was used as the control. HRP goat anti-rabbit IgG (H + L) (1:5000; ABclonal, AS014) was used as the secondary antibody. The primers used for mutagenesis analysis are listed in Additional file 1: Table S1.

### Feeding amount analysis

Newly molted fifth instar larvae were separated and reared on fresh mulberry leaves under standard conditions at 25 °C and 75% relative humidity. Mulberry leaves used for each feeding were placed in separate boxes as a control group. The single larva of each experimental group was fed with the same amount of fresh mulberry leaves in the blank control group. The try weight of the rest mulberry leaves weight in the control and experimental groups was recorded daily after oven drying at 80 °C for 4 h.

### Fecundity tests

Fecundity tests for male △*BmPiezo* were performed by mating single virgin males to WT virgin females for 4 h. Fecundity tests for female *BmPiezo* mutants were performed by mating single virgin females to WT virgin males for 4 h. The number of eggs laid by each female adult in each test was counted, and the hatching rate was determined 10–12 days later. The hatching rate (%) was determined by the percentage of eggs that gave viable progenies in the total number of oviposited eggs. The number of viable progenies was determined by counting the number of hatched larvae. For the detection of copulation success, each tested male was transferred to a 6-cm-diameter plate containing a WT virgin female. Copulation success was calculated as the percentage of tested males that mated with WT virgin females in 5 min. Copulation duration was measured as the percentage of tested males that mated with WT females for more than 4 h.

### RNA-seq analysis

Total RNA from the testes at pupal stages day 7 and the midgut at the third day of the fifth instar were extracted from WT and △*BmPiezo* with the methods described above. The cDNA libraries were generated by using the Illumina TruSeq™ RNA Sample Preparation Kit (Illumina, CA, USA) following the manufacturer’s recommendations. The cDNA libraries were then sequenced using the Illumina HiSeq 2000 platform (BGI, Wuhan, China). The raw data were qualified, filtered, and mapped to the silkworm genome database (http://kaikobase.dna.affrc.go.jp). Differentially expressed genes (DEGs) between WT animals and *BmPiezo* mutants were functionally annotated by GO and KEGG.

### Double copulation

In the control group, either a *BmPiezo* or a *BmSxl* mutant male was mated with a wild-type virgin female for 4 h. In the double-copulation group, a virgin female was first mated with the *BmPiezo* mutant male, followed by the *BmSxl* mutant male. After a single or double copulation, females were dropped in the chambers to lay eggs. The egg fertilization rate (%) was calculated as described above.

### Statistical analysis

The data were analyzed in GraphPad Prism 8. The normal distribution of the data was assessed using the Shapiro–Wilk tests. Experimental data were analyzed with the Fisher exact test, one-way ANOVA, or two-tail unpaired *t*-test. The homogeneity of variance of the data was assessed via the *F*-test. Normally distributed data with homogeneity of variance was analyzed with the two-tailed unpaired *t*-test or ANOVA (one-way ANOVA, Tukey’s multiple comparisons test). Normally distributed data with unequal variance were analyzed via the two-tailed unpaired *t*-test with Welch’s correction or Brown–Forsythe and Welch ANOVA tests (one-way ANOVA, Dunnett T3’s multiple comparisons test).

### Supplementary Information

**Additional file 1:** **Fig. S1** Phylogenetic analysis and structure prediction. **Fig. S2** The expression level of *BmPiezo* and loss of *BmPiezo* decreased defecation and body weight. **Fig. S3** Loss-of-BmPiezo leads to dysregulation of ribosome. **Fig. S4** Male copulation behavior. **Fig. S5** Representative confocal images of apyrene sperm bundles of WT and △*BmPiezo* from pupal stages day 7. **Fig. S6** Fertility is recovered by double copulation using △*BmSxl* and △*BmPiezo* male. **Table S1** Primers used in this work.

**Additional file 2:** **Video S1** Behavior of spermatozoa in the bursa copulatrix of females mated with WT males. Scale bar, 100 µm.

**Additional file 3:** **Video S2** Behavior of spermatozoa in the spermatheca of females mated with WT males. Scale bar, 100 µm.

**Additional file 4:** **Video S3** Behavior of spermatozoa in the bursa copulatrix of females mated with *∆BmPiezo* males. Scale bar, 100 µm.

**Additional file 5:** **Video S4** Behavior of spermatozoa in the spermatheca of females mated with *∆BmPiezo* males. Scale bar, 100 µm.

**Additional file 6:** The individual data values.

**Additional file 7:** Original western blot data.

## Data Availability

All data generated or analyzed during this study are included in this paper and its supplementary information files. The sequencing reads have been stored in the NCBI SRA database according to accession numbers (mRNA from testes at pupal stages day 7 of WT: SRR28840472, SRR28840475, SRR28840476; mRNA from testes at pupal stages day 7 of △*BmPiezo*: SRR28840469, SRR28840470, SRR28840471; mRNA from midguts at the third day of the fifth instar of WT: SRR28840466, SRR28840467, SRR28840468; mRNA from midguts at the third day of the fifth instar of △*BmPiezo*: SRR28840465, SRR28840473, SRR28840474).

## Abbreviations

P1: Day 1 of the pupal stage
q-RT-PCR: Quantitative real-time PCR
PBS: Phosphate-buffered saline
PBST: Phosphate-buffered saline, 0.1% Tween
TRITC: Tetramethylrhodamine isothiocyanate
