# Supplementary material for: The mechanoreceptor Piezo is required for spermatogenesis in Bombyx mori
Source: BMC Biol. 2024 May 20;22:118. doi: 10.1186/s12915-024-01916-y (PMC11106986; doi:10.1186/s12915-024-01916-y)
Supplement: Supplementary file 1 — Additional file 1: Fig. S1 Phylogenetic analysis and structure prediction. Fig. S2 The expression level of BmPiezo and loss of BmPiezo decreased defecation and body weight. Fig. S3 Loss-of-BmPiezo leads to dysregulation of ribosome. Fig. S4 Male copulation behavior. Fig. S5 Representative confocal images of apyrene sperm bundles of WT and △BmPiezo from pupal stages day 7. Fig. S6 Fertility is recovered by double copulation using △BmSxl and △BmPiezo male. Table S1 Primers used in this work. [file 12915_2024_1916_MOESM1_ESM.docx]

**The mechanoreceptor Piezo is required for spermatogenesis in *Bombyx mori***

Zhongjie Zhang^1,2*^, Xiaojing Liu^1,2^, Bo Hu^1,2^, Kai Chen^1,2^, Ye Yu^1,2^, Chenxin Sun^1,2^, Dalin Zhu^1,2^, Hua Bai^3^, Subba Reddy Palli^4^, Anjiang Tan^1,2*^

^1^Key Laboratory of Silkworm and Mulberry Genetic Improvement, Ministry of Agriculture and Rural Affairs, Jiangsu University of Science and Technology, Zhenjiang 212100, China.

^2^Jiangsu Key Laboratory of Sericultural Biology and Biotechnology, School of Biotechnology, Jiangsu University of Science and Technology, Zhenjiang 212100, China.

^3^Department of Genetics, Development, and Cell Biology, Iowa State University, Ames, IA 50011, USA.

^4^Department of Entomology, University of Kentucky, Lexington, KY 40546-0091, USA.

^*^Corresponding authors

E-mail: [zjzhang01@just.edu.cn](mailto:zjzhang01@just.edu.cn); [atan@just.edu.cn](mailto:atan@just.edu.cn.)


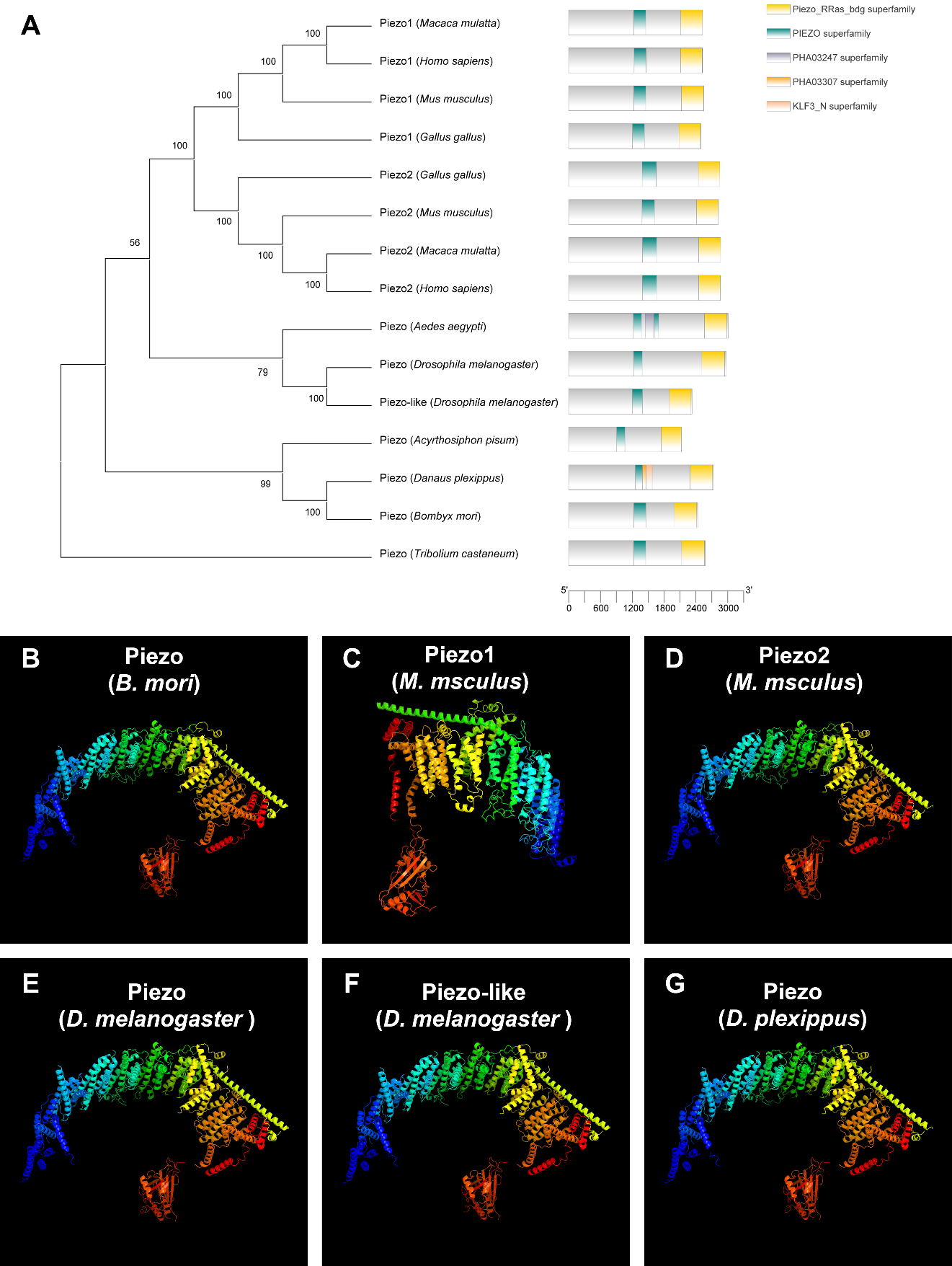


**Fig. S1 Phylogenetic analysis and structure prediction.** (A) Maximum likelihood tree of Piezo. The accession numbers of the protein sequences were as follows: *Drosophila melanogaster* Piezo (NP_001303314.1), Piezo-like (NP_001303493.1); *Aedes aegypti* (XP_021703538.1); *Mus musculus* Piezo1 (NP_001032375.1), Piezo2 (NP_001034574.4); *Tribolium castaneum* Piezo (XP_015835784.1); *Gallus gallus* Piezo1 (XP_040537376.1), Piezo2 (XP_040520788.1); *Macaca mulatta* Piezo1 (XP_028696932.1), Piezo2 (XP_028693994.1); *Homo sapiens* Piezo1 (NP_001136336.2), Piezo2 (NP_001365112.1); *Danaus plexippus* Piezo (XP_061378195.1); *Acyrthosiphon pisum* Piezo (XP_016660213.2). The conserved domains were identified and showed on the right. (B-G) Comparison of predicted three-dimensional structure of *B. mori* Piezo, *M. musculus* Piezo1, *M. musculus* Piezo2, *D. melanogaster* Piezo, *D. melanogaster* Piezo-like and *D. plexippus* Piezo.


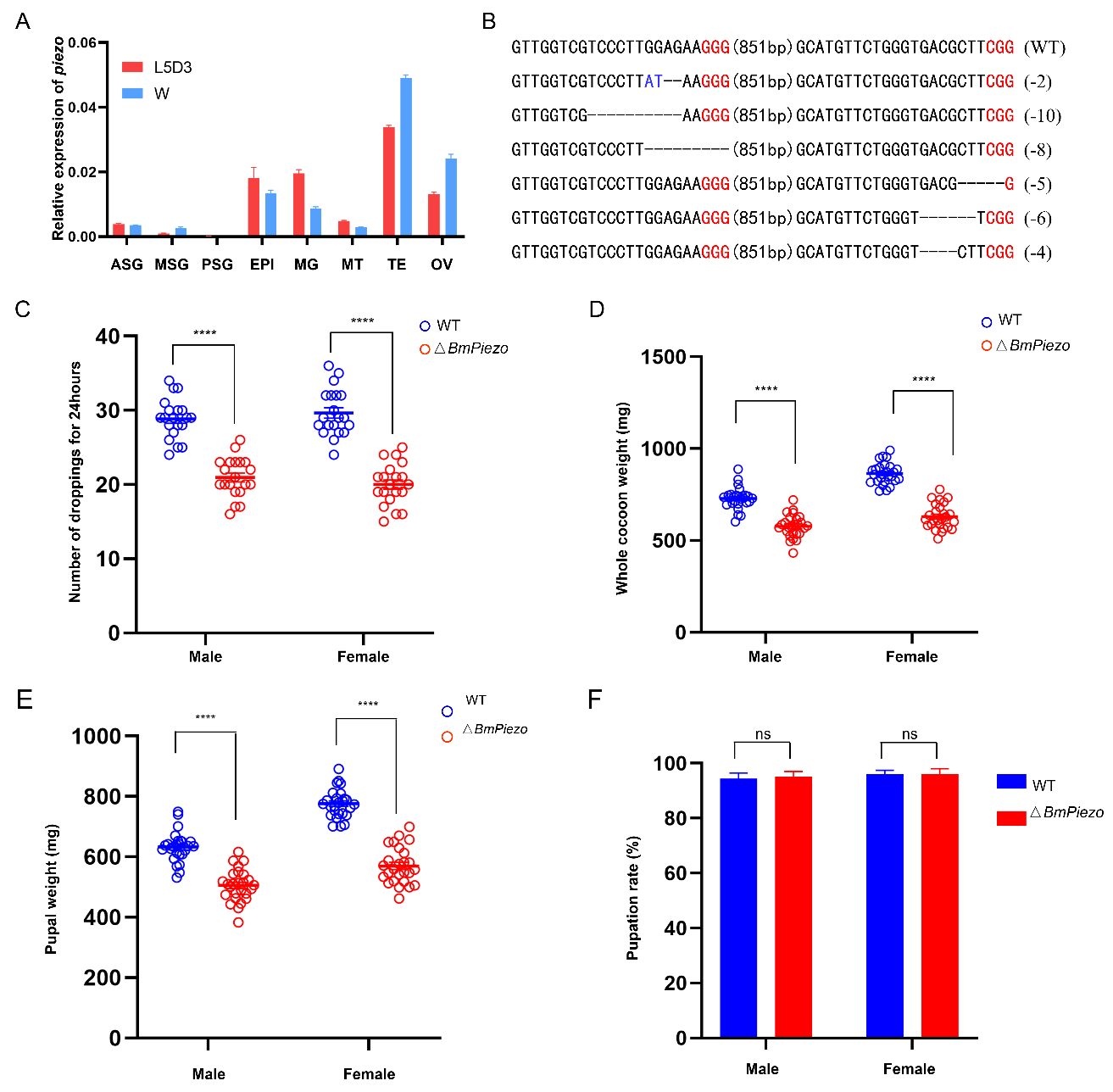


**Fig.** **S2 The expression level of *BmPiezo* and loss of *BmPiezo* decreased defecation and body weight.** (A) The relative transcript level of BmPiezo in various tissues from day 3 of the fifth instar (L5D3) and wandering stage (W). Tissues including anterior silk gland (ASG), middle silk gland (MSG), posterior silk gland (PSG), epidermis (EP), midgut (MG), malpighian tubule (MT), testes (TE) and ovary (OV). (B) Sequence of mutations at TS1 and TS2 induced by CRISPR/Cas9. The dashes in every sequence line represent deleted residues and the detail indel size is shown on the right. The base of point mutation is shown in blue. (C) Number of droppings (per larva) from newly molted fifth larvae fed on mulberry at 24 hours after initiation of feeding. The data are shown as the mean ± SEM (n = 20). ****, p < 0.0001 by two-tailed unpaired *t* test. (D) The whole cocoon weight of WT and △*BmPiezo*. The data are shown as the mean ± SEM (n = 26). ****, p < 0.0001 by two-tailed unpaired *t* test. (E) The pupal weight of WT and △*BmPiezo*. The data are shown as the mean ± SEM (n = 26). ****, p < 0.0001 by two-tailed unpaired *t* test. (F) The pupation rate of WT and △*BmPiezo*. The data are shown as the mean ± SEM (n = 6). ****, p < 0.0001 by two-tailed unpaired *t* test.


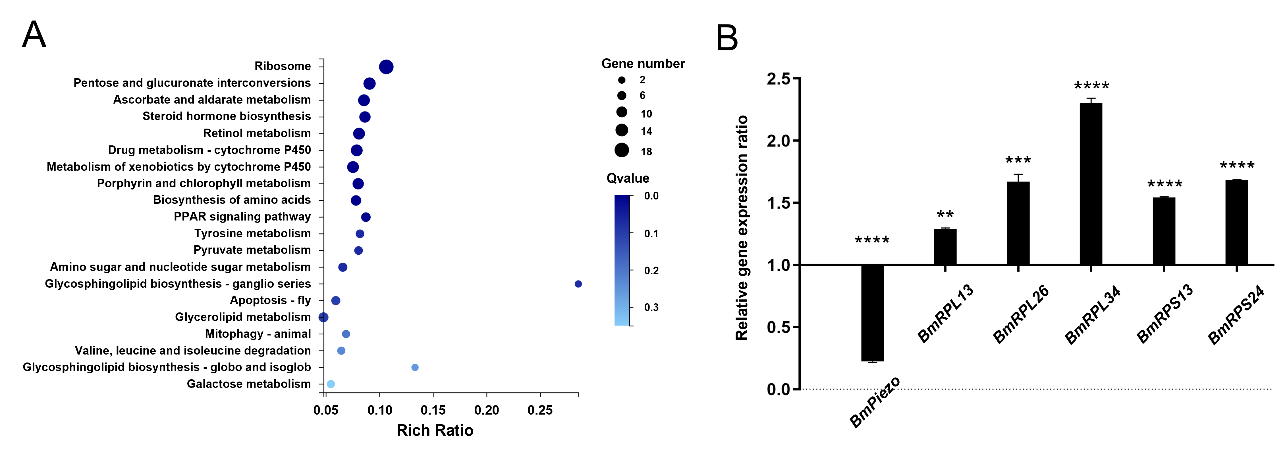


**Fig. S3 Loss-of-*BmPiezo* leads to dysregulation of ribosome.** (A)The top 20 enriched Kyoto Encyclopedia of Genes and Genomes (KEGG) pathways of DEGs with p < 0.05. (B) Validation ofRNA-Seq revealed gene expression changes in ribosome by qRT-PCR. The results were measured in triplicate and are shown as mean ± SEM. The asterisks indicate the significant differences compared with the relevant control with two-tailed unpaired *t*-test. *P < 0.05; **P < 0.01; ***P < 0.001; ****P < 0.0001.


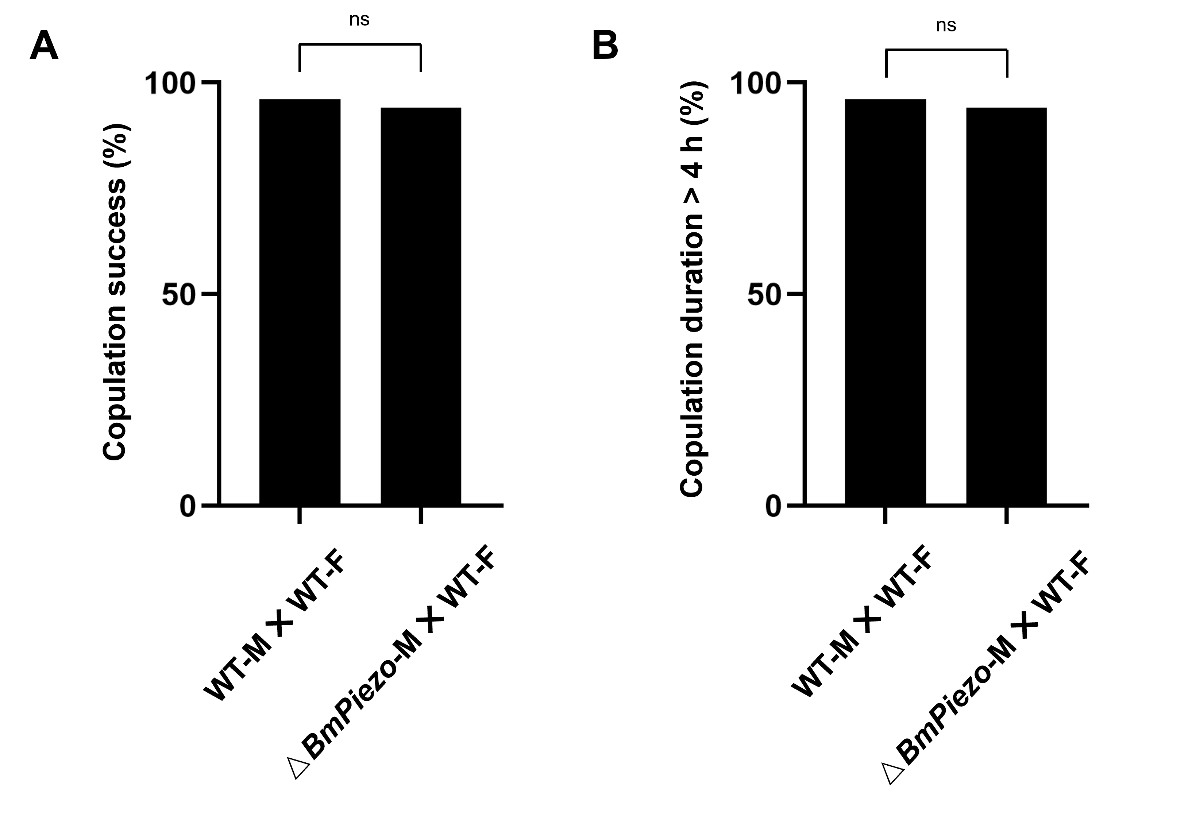


**Fig. S4 Male copulation behavior.** (A) Copulation success (n = 50, p ≥ 0.05, Fisher exact test). (B) Copulation duration (n = 45, p ≥ 0.05, Fisher exact test).


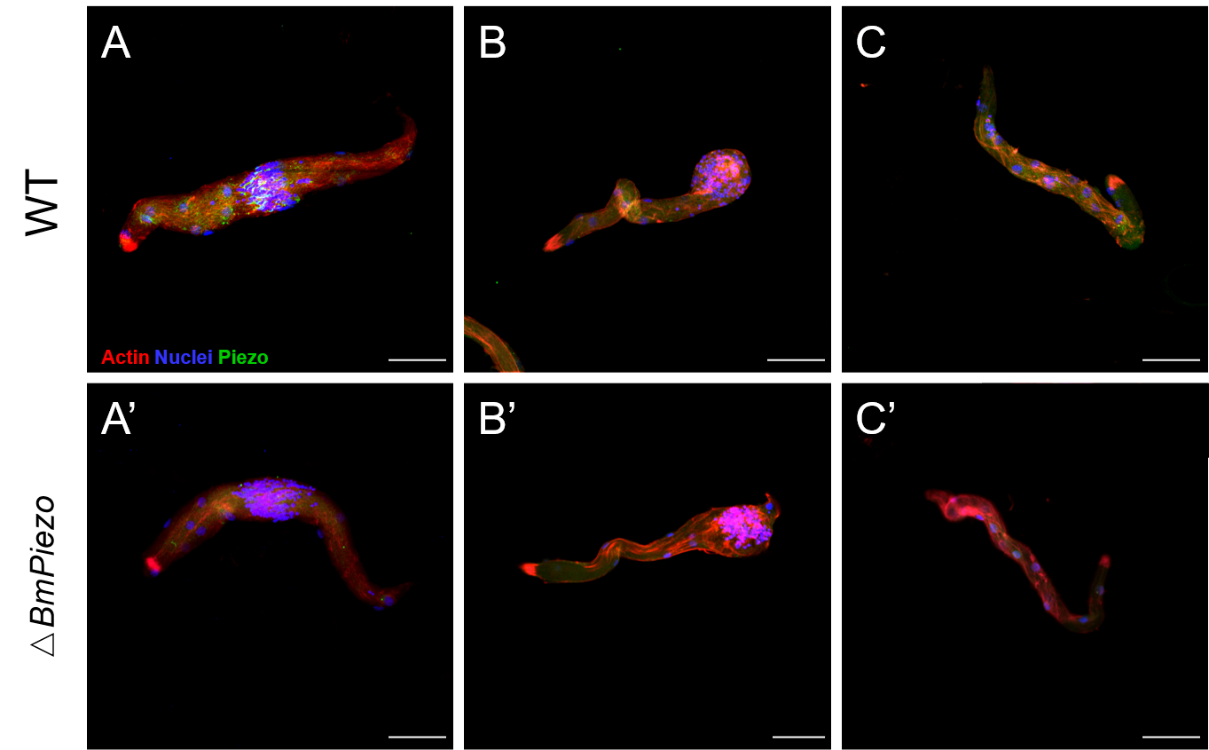


**Fig.** **S5 Representative confocal images of apyrene sperm bundles of WT and △*BmPiezo* from pupal stages day 7**. (A, A’) Elongating apyrene sperm bundles with distribution of small round micronuclei in the middle region. (B, B’) Squeezed apyrene sperm bundles with distribution of small round micronuclei in the posterior region. (C, C’) Mature apyrene sperm bundles without small round micronuclei. Blue: Hoechst; Red: filamentous actin (F-actin), Green: anti-BmPiezo. Scale bars: 50 μm.


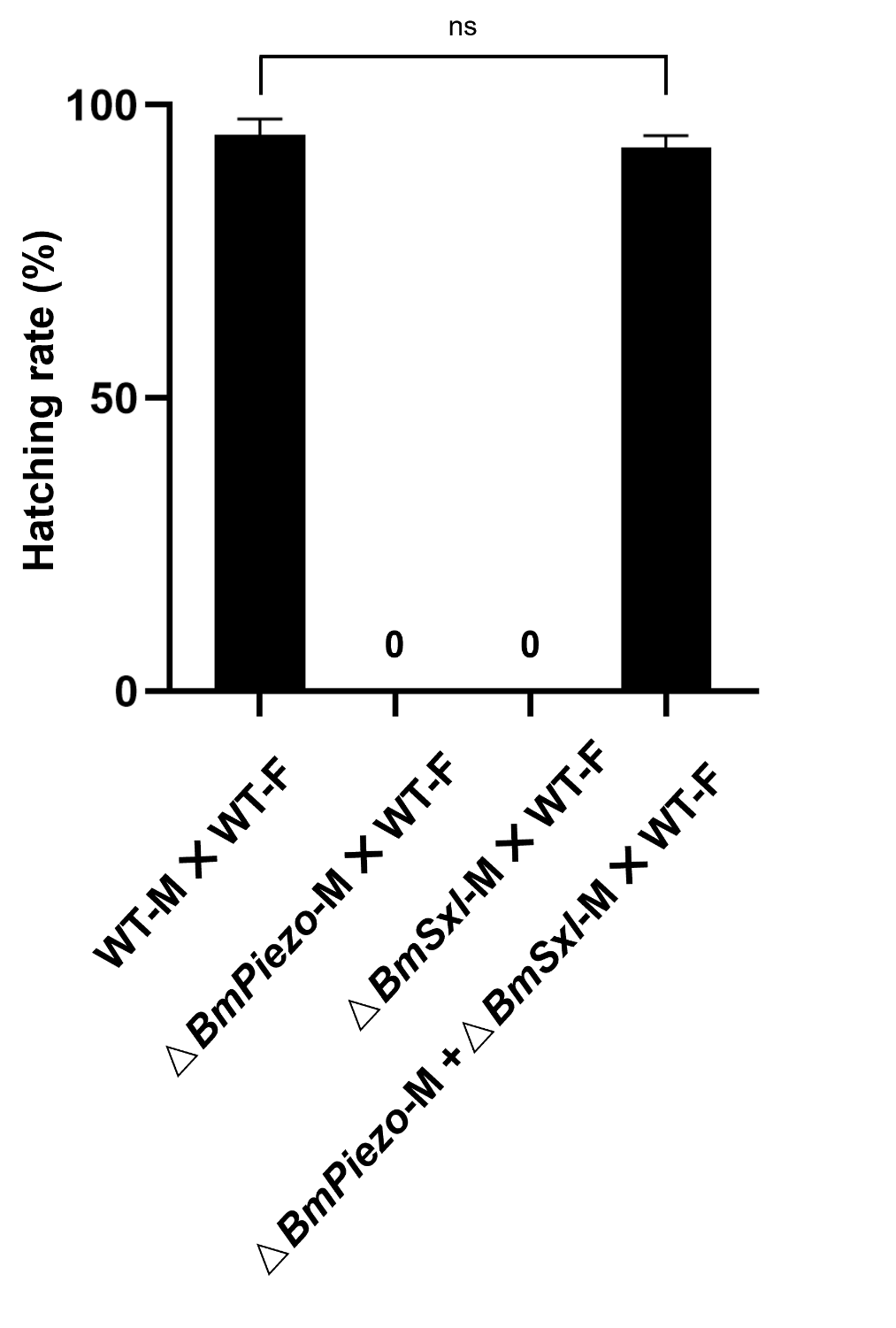


**Fig. S6 Fertility is recovered by double copulation using △*BmSxl* and △*BmPiezo* males.** Hatch rates were analyzed in single or double copulation with WT females. The horizontal axis shows the genotypes of males used in copulation tests. Target females were wild type. The results are expressed as hatching rate from 10 broods tests with two-tailed unpaired *t* test. An index of 0% indicates the absence of newly hatched larvae.

**Table S1. Primers used in this work.**

| Primer name | Primer sequence (5’–3’) | Primer purpose |
| --- | --- | --- |
| HR-F | TCACTATAGGGCGAATTGGAGGTTATGTAGTACACATTGTTGTA | Plasmid construction |
| U6-R1 | TTCTCCAAGGGACGACCAACACTTGTAGAGCACGATATTTTGTAT |  |
| sgRNA-F1 | GTTGGTCGTCCCTTGGAGAAGTTTTAGAGCTAGAAATAGCAAGT |  |
| Overlap-R | CCGCGGAGTCAATGGCTAGCAAAAAAGCACCGACTCGGTG |  |
| Overlap-F | GCTAGCCATTGACTCCGCGGAGGTTATGTAGTACACATTGTTGTA |  |
| U6-R2 | AAGCGTCACCCAGAACATGCACTTGTAGAGCACGATATTTTGTAT |  |
| sgRNA-F2 | GCATGTTCTGGGTGACGCTTGTTTTAGAGCTAGAAATAGCAAGT |  |
| HR-R | TTTCTTGTTATAGATATCAAAAAAAGCACCGACTCGGTG |  |
| *BmPiezo*-TS1F | CACATAGACTGCACAATCCGAC | Mutation detection |
| *BmPiezo*-TS1R | CACACCTACGAGTAGTGTATCG |  |
| *BmPiezo*-TS2F | CGTTACGTAGAAAGGGCAAGTG |  |
| *BmPiezo*-TS2R | AGGAGACGATACAGTCAGAGTC |  |
| *BmPiezo*-F | TCACCAGCTCTGTGGTTCAG | qRT-PCR |
| *BmPiezo*-R | CCATTCTGCGTTGTTGTACG |  |
| *BmIntegrin* α-F | AATTTCGGGTACAGCGTGAC |  |
| *BmIntegrin* α-R | ATACGAGCCGAGCACAAACT |  |
| *BmTensin*-F | CGCCTTCCTCAAGATCTACG |  |
| *BmTensin*-R | AACTGACACGCGAACACAAG |  |
| *BmVEPTP*-F | CGGAGTCTTTGGGTTTCAGT |  |
| *BmVEPTP*-R | GTACGCGGCTTTGTTTCATT |  |
| *BmTenascin-like*-F | GCGTTTGCGATAATTTCCTC |  |
| *BmTenascin-like*-R | CGCATTGTCCACCAATACAG |  |
| *BmHemicentin -2*-F | CATCGGCGTTTCAATGTATG |  |
| *BmHemicentin -2*-R | GATCTTCATCGCTCCGACTC |  |
| *BmRPL12*-F | TAGCTGTTGTTCCCTCTGCA |  |
| *BmRPL12*-R | TACCGGGCCATTGATCTGTT |  |
| *BmRPL26*-F | GAGAAAAGGCCAATGGTGCA |  |
| *BmRPL26*-R | TGGCAGTTTCCTCGGTGTAT |  |
| *BmRPL34*-F | GCTTACATTCAGGCGACGAC |  |
| *BmRPL34*-R | TACGGTAGCAAAGACGGGAA |  |
| *BmRPS13*-F | ACTGCCGACGATGTAAAGGA |  |
| *BmRPS13*-R | TCAGGAGCTAGACCCATTGC |  |
| *BmRPS24*-F | TGAGTGAAGGAACAGCGACT |  |
| *BmRPS24*-R | AGCGAATCCAGTTGACTTGC |  |
