# Supplementary figures and images for: The mechanoreceptor Piezo is required for spermatogenesis in Bombyx mori
Source: BMC Biol. 2024 May 20;22:118. doi: 10.1186/s12915-024-01916-y (PMC11106986; doi:10.1186/s12915-024-01916-y)

**Figure 1B**


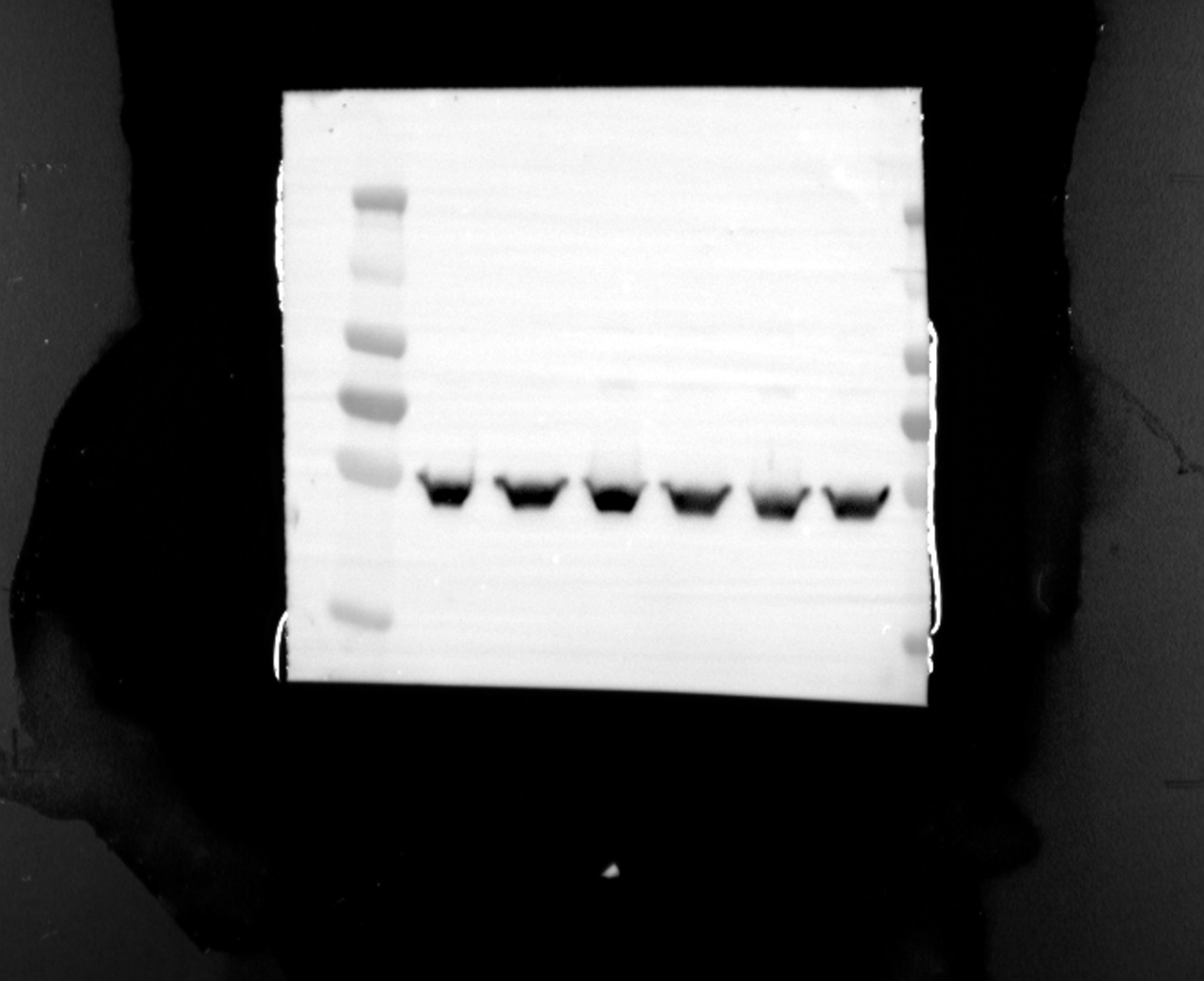




**β-Tubulin**

**BmPiezo**

Supplement: Supplementary file 7 — Additional file 7: Original western blot data. [file 12915_2024_1916_MOESM7_ESM.docx]
